# Inequalities in Cardiovascular Health Between Local and Migrant Residents: A Cross-Sectional Study of 6934 Participants in Longhua District, Shenzhen

**DOI:** 10.1097/MD.0000000000002103

**Published:** 2015-12-11

**Authors:** Weikang Yang, Haitao Li, Xiaoyuan Fu, Junqiang Lu, Zhiqiang Xue, Chuan’an Wu

**Affiliations:** From the Health Education Institute of Longhua New District, Shenzhen, P.R. China (WY, ZX, CAW); and School of Medicine, Shenzhen University, Shenzhen, P.R. China (HL, XF, JL).

## Abstract

Household registration status is one social determinant that influences health disparities. This study aimed to investigate the disparities in cardiovascular health between local and migrant residents, which may provide important implications for public health services and may help improve cardiovascular health for residents in Shenzhen.

A cross-sectional study was conducted in Shenzhen City Longhua district. Participants were selected for face-to-face interview surveys by using a multistage cluster random sampling design. Chi-square tests and multiple logistic regression models were constructed to compare cardiovascular health between the migrant and local residents.

A total of 6934 eligible respondents, of whom 1400 were local and 5534 were migrants, completed the face-to-face interview surveys. The local residents were more likely to have hypertension (3.1% vs. 2.0%, *P* < 0.05) and diabetes mellitus (1.4% vs. 0.5%, *P* < 0.05), whilst to be overweight or obese (20.3% vs. 16.4%, *P* < 0.05) when compared with their migrant counterparts. A higher proportion of local residents than migrant ones had ≥2 cardiovascular risk factors, 2.4% and 1.2%, respectively (*P* < 0.01). Compared with migrants, the locals were more likely to know their BP values (65.4% vs. 54.5%, *P* < 0.05) and know the symptoms of diabetes (63.1% vs. 49.7%, *P* < 0.01).

Our study suggests that household registration status is an important driver of social disparities in cardiovascular health except for the factors regarding socioeconomic status. Programs to improve the awareness of hypertension and diabetes are suggested to be initiated among the migrants.

## INTRODUCTION

Cardiovascular disease (CVD) is an alarming sign throughout the world. Statistics showed that CVD was the leading cause of death globally, causing about 17 million deaths (31.5%) in 2013.^1^ It is estimated that by 2020, CVD will claim 25 million lives each year and will surpass infectious diseases as the leading contributor to the global disease burden.^2^ In China, CVD is estimated to account for approximately 38% of total mortality.^3^ CVD events are predicted to increase by 23% by 2030, resulting in an additional 21 million CVD events and 7.7 million deaths.^4^ Common risk factors for CVD have been identified including hypertension, diabetes, overweight, obesity, etc. In China, the prevalence of hypertension reached 34% among adults aged ≥25-year-old in 2011.^5^ The prevalence rate was estimated to be 11.6% for diabetes mellitus in Chinese adult population,^6^ and 23.2% for overweight and obesity.^7^ The prevalence of diabetes in China has increased rapidly over the past few decades with a disproportionate burden among young and middle-aged individuals.^8^ Similarly, the increase trend in the prevalence of hypertension is much greater in younger age groups than in older age groups.^5,9^ CVD in the youth causes much greater economic and societal burden than that in the elders, raising concern of policy makers in China for the greater increase of prevalence of CVD in the youth than in the elders as a consequence of greater increase of prevalence of hypertension and diabetes in the youth.

It is widely recognized that social determinants of health are a major source of health disparities.^10,11^ The inverse association of socioeconomic status with various CVD risk factors, such as hypertension, diabetes, and obesity has been well established.^12–16^ The relationship between psychosocial factors and CVD risk factors has been identified too.^17–19^ Household registration status is also one social determinant that influences health disparities. Previous studies have documented a general trend toward a positive relationship between migration and poor quality of health care received and the following poor health outcomes.^20–23^

In China, *Hukou* refers to a household registration status which collapses people into 2 categories, that is, internal migrants and local residents.^20^ China has a large number of internal migrants—nearly 230 million by the end of 2011.^24^ Shenzhen, a major city situated in the Pearl River Delta region of China, has millions of internal migrants.^25^ Statistics showed that approximately 80% of Shenzhen population were internal migrants, reaching nearly 12.6 million in 2012.^26^ Studies suggested that these internal migrants usually have poorer sociodemographic characteristics such as lower income and education level when compared with their local counterparts. They also usually have poorer access to health care due to low payment ability of health services and a lack of health insurance, resulting in unsatisfactory health outcomes.^20,27^ Studies have also suggested that Chinese migrants have an excess smoking prevalence.^28,29^ Alcohol intoxication is reported to be elevated among migrants too.^30^ We thus assumed a higher prevalence of CVD risk factors among migrants than among local residents.

Previous studies have consistently demonstrated that migrants have a better self-reported health status and lower incidence of acute illnesses, chronic diseases, and disabilities than local residents.^31–33^ Whether conclusions of previous studies are applicable to CVD health is largely unknown. To understand the differences in CVD health between migrant and local residents has important implications for public health services. This study investigated the disparities in CVD health between local and migrant residents in Longhua District, Shenzhen, which may provide clues about the etiology of CVD health and may help improve CVD health for residents in Shenzhen.

## METHODS

### Sampling Frame and Procedures

A cross-sectional study was conducted in Longhua district, Shenzhen City in 2014. Employing a multistage cluster random sampling method, individuals were selected for face-to-face interview survey. The sampling involved 3 stages. Firstly, 4 geographic areas, namely Longhua, Minzhi, Dalang, and Guanlan, were divided. Two neighborhoods were then randomly selected in each geographic area by using simple random sampling methods. A total of 8 neighborhoods were chosen. At last, we used systematic random sampling methods to select households on the basis of household roster.

The target population consisted of all residents in the selected communities. With an estimated proportion difference of (4.0–2.0%) for poor CVD health,^34^ we calculated the minimum sample size of 1427 was needed to generate a 99% confidence interval and 90% statistical power. In Shenzhen, the number of migrants was almost 4 times that of the locals, on the basis of which we calculated the estimated sample size for the migrants (5707). We then planned to select 905 households from each neighborhood, giving 7240 households in total. Inclusion criteria of the respondents were age between 15 and 69 years, ability to communicate and give informed consent, and last birthday closest to the date of the interview (to minimize over-representation of housewives and the elderly). Face-to-face interview surveys were performed by extensively trained interviewers from the Guangdong Pharmaceutical University. The respondents were assured of the anonymity and confidentiality of the survey, and informed consent was obtained before the surveys commenced. At last, a total of 6934 respondents finished the survey, with a response rate of 96%. Ethical approval was obtained from the Longhua District Health Education Institute Ethics Committee.

### Key Measures

The main outcome of interest was poor cardiovascular health. We defined ≥2 self-reported CVD clinical risk factors as poor cardiovascular health. Clinical risk factors were based on respondents who answered “yes” to the question of ever being told by a doctor, nurse, or other health professional that they had diabetes and hypertension. Obesity status was also included as a clinical risk factor based on self-reported height and weight. Individuals with a BMI ≥24.0 were considered overweight and obese and at greater risk for CVD.^35^

The socioeconomic characteristics of respondents were collected including household register, education level, employment status, and monthly income. The distribution of these categorical variables was evaluated, and appropriate cut-points were determined by the distribution of variables and also based on prior literature. According to household register, respondents were grouped into local residents and migrants. Migrants were internal migrants which refer to those who move to the new location without changing their official *Hukou* registration. Education level was collapsed into 3 categories: less than high school, high school diploma and equivalent, and 3-year college and above. Employment status was classified into 2 categories: those who have a job (including employed and self-employed) and those who do not have a job (including students, the retired, housewives, and the unemployed). We allocated the respondents into 3 economic groups according to monthly poverty line and mean income level in Shenzhen in 2011, that is, below RMB 2000 (low income group), between RMB 2000 and 5000 (middle income group), and above RMB 5000 (high income group).^36^ The demographic factors were also collected including age and gender. Other covariates included smoking status (Yes/No), drinking (Safe or Not), and exercising habits (Regularly, Irregularly, and No). Smokers referred to those who smoked at least 100 cigarettes in their lifetime and were currently smoking. Safe drinking referred to the everyday intake of alcohol less than 20 ml for men, while 10 ml for women.^37^ The respondents with regular physical exercise were those who did exercise at least three times per week with each time no less than 30 minutes.

Additionally, to understand the respondents’ knowledge on hypertension and diabetes, we asked some questions including awareness of their blood pressure (BP), as well as what kind of disease was indicated by symptoms of frequent urination, increased thirst, and increased hunger.

### Statistical Analysis

All collected data were input into the EpiData 3.1 database. Descriptive statistics were presented regarding the respondents’ sociodemographic characteristics, CVD outcomes, behavioral factors, knowledge of hypertension, and diabetes. The Chi-square tests were performed to examine the differences in the sociodemographic characteristics and behavioral factors between the locals and migrants. Both Chi-square tests and multiple logistic regression models were employed to examine whether or not significant differences in CVD outcomes and knowledge of hypertension and diabetes existed between the locals and their migrant counterparts. The confounding variables in the multiple regression analysis included all sociodemographic characteristics (age, gender, occupation, education, and income) and behavioral factors (smoking status, drinking, and exercising habits). For all tests performed in the study, a *P*-value of less than 0.05 was adopted as the statistically significant level. All analyses were conducted using SPSS 19.0.

## RESULTS

A total of 6934 eligible respondents, of whom 1400 were local and 5534 were migrants, completed the face-to-face interview surveys. A higher proportion was female (58.1%), and the majority was aged 44 years or younger (93.3%). Most of the respondents reported that they had a job (95.7%). The respondents were approximately equally classified into three education groups. Around two-thirds (61.0%) had middle income level. Approximately 19.0% respondents were smokers, 74.9% were individuals with safe drinking habits, while only 12.8% did exercise regularly. There was significant heterogeneity between local and migrant residents in terms of all respondents’ characteristics except for occupation status (Table 1).

**TABLE 1:**
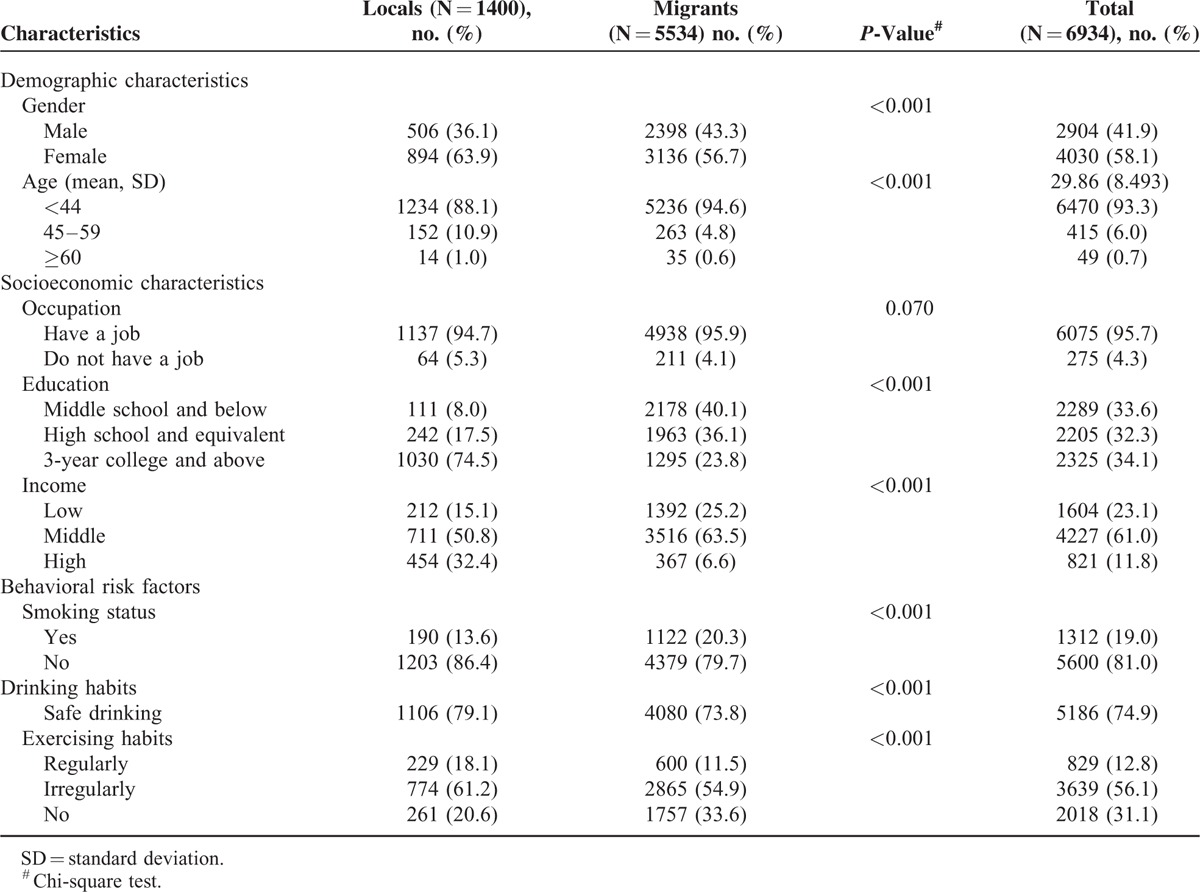
Sociodemographic Characteristics and Behavioral Risk Factors of the Respondents

The local residents were more likely to have hypertension (3.1% vs. 2.0%, *P* = 0.015) and diabetes mellitus (1.4% vs. 0.5%, *P* = 0.001), whilst to be overweight or obese (20.3% vs. 16.4%, *P* = 0.001) when compared with their migrant counterparts. A higher proportion of local residents than migrant ones had ≥2 CVD risk factors, 2.4% and 1.2%, respectively (*P* = 0.001). After adjustments were made for respondents’ characteristics, these significant differences sill remained (Table 2).

**TABLE 2:**
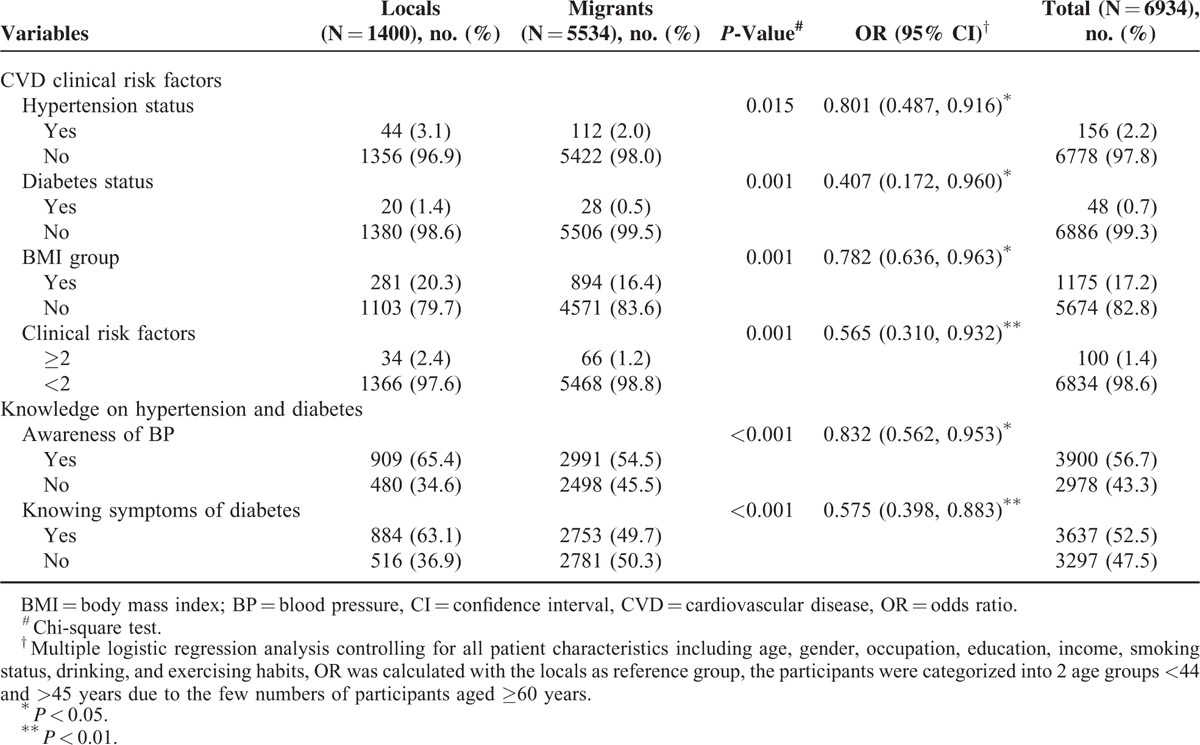
The Respondents’ Cardiovascular Health and Their Knowledge on Hypertension and Diabetes

Compared with migrants, the locals were more likely to know their BP values (65.4% vs. 54.5%, *P* < 0.001) and know the symptoms of diabetes (63.1% vs. 49.7%, *P* < 0.001). After adjusting for respondents’ characteristics, similar findings were found (Table 2).

## DISCUSSION

Our study found that the proportions of respondents who had hypertension and diabetes were higher among the locals than among the migrants. Meanwhile, the locals were more likely to be overweight or obese when compared with the migrants. Overall, the locals tended to have worse CVD health than the migrants. Furthermore, we found that the locals were more likely to know their BP values and to know the symptoms of diabetes.

It was found that the locals were more likely to have worse CVD health than their migrant counterparts. Our study showed that the locals tended to have a higher education level and better income when compared with the migrants. This is in contrast to previous studies that found inverse association between socioeconomic status and CVD health. The study by Sharon and colleagues^34^ found a graded inverse association between socioeconomic status and CVD health regardless of race and location. Studies have also established inverse association of socioeconomic status with hypertension, diabetes, and obesity.^12–16^ One possible explanation of the finding was the lower awareness rate of hypertension and diabetes in migrant residents than in the locals. Our study found that more local residents were aware of their BP values, whilst got to know the symptoms of diabetes compared with the migrants. Therefore, the lower awareness rate of hypertension and diabetes and the followed lower reporting rate may explain the worse CVD health in local residents than in the migrants.

The lower awareness rate of BP values in the migrants than in the locals may be due to the differences in the health insurance schemes between the locals and migrants. Health insurance coverage rate is significantly higher among the locals than the migrants,^22^ although Shenzhen government has taken actions to improve health insurance coverage rate in the migrants. For example, Shenzhen government initiated its resident card system in 2008 which ensures legitimacy for migrants to join the city's Comprehensive Health Insurance Scheme as an individual except for the Medical Insurance Scheme for Migrant Employees as employed migrants. Studies have shown a positive association between health insurance coverage and access to and utilization of healthcare services.^25^ Insufficient health insurance coverage among the migrants poses barriers when they seek health services. Additionally, reimbursement constraint for those under Medical Insurance Scheme for Migrant Employees may result in them seeking health care primarily for acute problems.^38^ The lower access to and utilization of healthcare services may thus lead to a lower awareness rate of hypertension status in the migrants than in the locals.

It was shown that the locals were more likely to be overweight or obese compared with the migrants, which deepens the gap in the CVD health between the locals and the migrants. Migrants are generally less skilled and minimally educated, and hence tend to be manual workers.^27^ Their occupational physical exercise is thus more common than that of the locals.^39^ Moreover, the consumption of animal protein and the proportion of calories derived from fat are higher in the locals than in the migrants, which is possibly due to the lower economic capacity of the migrants.^40^ The fact that western diet is related to the increased CVD events is well established. Therefore, in view of diet and physical exercise, the prevalence of overweight and obesity in the locals is higher than that in the migrants.

Results showed that the prevalence of hypertension was only 2.2% which is much lower than that in the general population across Shenzhen (about 15%). One possible reason of this finding is different structures between the 2 populations. The mean age of the respondents in the present study was 29.86 years, while the mean age of the residents across Shenzhen was 33.6 years in 2013. Moreover, the subjects aged ≥60 years accounted for only 0.7% of total respondents which is much lower than that of the general population across Shenzhen (3.29%). It has been shown that hypertension is the commonest comorbidity among the elderly,^41^ and its prevalence increases with age.^42,43^ Another possible explanation of the finding is the low awareness rate of hypertension. The 2002 national hypertension survey in China showed that only 23% patients with hypertension were aware of it.^44^ Our study also showed that only about half of the respondents knew their BP values. Similar observations were found for diabetes.

The limitations of the study should be considered. Firstly, the study sample was selected from one district of Shenzhen city; therefore, the findings cannot be generalized to all residents across Shenzhen. Secondly, the measures of CVD health including height, weight, hypertension, and diabetes were self-reported which are prone to recall bias, although previous studies have demonstrated the accuracy of self-report of cardiovascular history.^45^ Thirdly, we only used BMI, hypertension and diabetes to measure CVD health, but not included indicators such as cholesterol level, which may bias the findings of the present study. Additionally, other potential confounding variables such as societal stress, rural/urban status, residential region of the home address (western, eastern, or central China) were not collected in the present study; these factors were not controlled in the analysis. Fourthly, the participants were simply classified into 2 categories regarding employment status, since there were only a small number of students and the retired. Statistical power of multiple regression analysis may be reduced due to the heterogeneity among students, the retired, housewives, and the unemployed. Finally, the cross-sectional nature of our study warrant further investigations to establish causal inferences between household registration status and CVD health.

Our study suggests that household registration status is an important driver of social disparities in CVD health in Shenzhen except for the factors regarding socioeconomic status. CVD risk reduction interventions should be implemented among the local residents in Shenzhen. Policy makers may consider initiating programs to expand access to treatment and management of hypertension and diabetes. As for the migrants, how to improve their awareness of hypertension and diabetes should be in the priority. Free health examinations including measurement of BP has been suggested to be offered to all residents by primary care providers, but the utilization rate is low due to the perceived low quality of services provided by primary care physicians. The national program with respect to the establishment of health records for all residents may be a favorable approach for the detection of hypertension and diabetes; the information should be updated periodically such as 1 year. Health education should be provided effectively to the community residents especially the migrants. The Ministry of Health has announced a project in 2010 that aims to provide healthcare services and health education to migrant workers in 65 major cities and counties in 29 provinces. The adoption of effective approaches to attract migrants to join the health education programs should be considered by policy makers. Future studies may try to establish causal inferences between household registration status and CVD health, and to investigate the possible pathways causing the disparities.
